# Three-year follow-up of canakinumab dose extension in children with colchicine-resistant familial Mediterranean fever: PeRA-RG Experience

**DOI:** 10.1093/rheumatology/keaf403

**Published:** 2025-08-01

**Authors:** Gülşah Kavrul Kayaalp, Şengül Çağlayan, Kadir Ulu, Şeyma Türkmen, Fatma Gül Demirkan, Vafa Guliyeva, Gülçin Otar Yener, Kübra Öztürk, Ferhat Demir, Semanur Özdel, Mustafa Çakan, Hafize Emine Sönmez, Betül Sözeri, Nuray Aktay Ayaz, the PeRA Research Group, Gülşah Kavrul Kayaalp, Şengül Çağlayan, Kadir Ulu, Şeyma Türkmen, Fatma Gül Demirkan, Vafa Guliyeva, Gülçin Otar Yener, Kübra Öztürk, Ferhat Demir, Semanur Özdel, Mustafa Çakan, Hafize Emine Sönmez, Betül Sözeri, Nuray Aktay Ayaz

**Affiliations:** Department of Pediatric Rheumatology, Istanbul University, Istanbul Faculty of Medicine, Istanbul, Turkey; Department of Pediatric Rheumatology, Ümraniye Research and Training Hospital, Istanbul, Turkey; Department of Pediatric Rheumatology, Antalya Research and Training Hospital, Antalya, Turkey; Department of Pediatric Rheumatology, Ümraniye Research and Training Hospital, Istanbul, Turkey; Department of Pediatric Rheumatology, Şehit Prof. Dr İlhan Varank Sancaktepe Research and Training Hospital, Istanbul, Turkey; Department of Pediatric Rheumatology, Ümraniye Research and Training Hospital, Istanbul, Turkey; Department of Pediatric Rheumatology, Istanbul University, Istanbul Faculty of Medicine, Istanbul, Turkey; Department of Pediatric Rheumatology, Istanbul University, Istanbul Faculty of Medicine, Istanbul, Turkey; Department of Pediatric Rheumatology, Sanliurfa Research and Training Hospital, Sanliurfa, Turkey; Department of Pediatric Rheumatology, Goztepe Prof. Dr Süleyman Yalçın Research and Training Hospital, Istanbul Medeniyet University, Istanbul, Turkey; Department of Pediatric Rheumatology, Acıbadem Health Groups Hospital, Istanbul, Turkey; Department of Pediatric Rheumatology, Ankara Dr Sami Ulus Research and Training Hospital, Ankara, Turkey; Department of Pediatric Rheumatology, Zeynep Kamil Research and Training Hospital, Istanbul, Turkey; Department of Pediatric Rheumatology, Kocaeli University Faculty of Medicine, Kocaeli, Turkey; Department of Pediatric Rheumatology, Ümraniye Research and Training Hospital, Istanbul, Turkey; Department of Pediatric Rheumatology, Istanbul University, Istanbul Faculty of Medicine, Istanbul, Turkey

**Keywords:** Familial Mediterranean fever, canakinumab, anti-interleukin-1 therapy, colchicine resistance

## Abstract

**Objectives:**

Anti-interleukin-1 therapies are effective for colchicine-resistant FMF, yet data on optimal duration in pediatric patients remain limited. A previous multicentre study showed favourable outcomes with a standardized canakinumab dose extension protocol, though follow-up was short. This study aimed to assess the long-term outcomes of this protocol.

**Methods:**

The protocol, developed via a multicentre Delphi consensus in Turkey, recommends doubling the canakinumab dosing interval after 6 attack-free months and tripling it after 1 year of continued remission. This retrospective study included colchicine-resistant FMF patients treated according to the protocol, with data extracted from medical records.

**Results:**

Forty-five patients initiated monthly canakinumab. The median follow-up after starting canakinumab was 47 months (range 35–78). After 6 months, intervals were extended to every 2 months. During bimonthly dosing, 7 patients (15.6%) experienced attacks and reverted to monthly dosing. Of the 38 patients (84.4%) whose interval was further extended to every 3 months, 11 returned to bimonthly dosing due to attacks. Among 10 patients who achieved remission with 3-month intervals, treatment was discontinued; 5 remained attack-free, while 5 had attacks. Seventeen patients continued 3-monthly dosing, 1 was lost to follow-up and 16 (35.6%) remained attack-free at a median follow-up of 33 months (interquartile range: 6.5). Clinical and laboratory findings were similar between patients with and without attacks, except splenomegaly, which was absent in the attack-free group (*p* = 0.006).

**Conclusion:**

The dose extension protocol shows promising long-term outcomes in colchicine-resistant FMF. Larger, prospective studies are warranted to optimize treatment strategies.

Rheumatology Key messages •  The standardized canakinumab dose extension protocol appears promising and may be considered for some patients.

•  Canakinumab remains effective with extended dosing intervals in some patients, irrespective of baseline attack features and laboratory values.

•  Canakinumab dosing intervals could not be extended in patients with splenomegaly at baseline.

## Introduction

FMF is the most common hereditary autoinflammatory disease predominantly seen in populations from the Mediterranean region. Characterized by recurring episodes of fever and serositis, FMF is associated with mutations in the *MEFV* gene, which disrupt inflammasome regulation and lead to increased IL-1 production [1–3]. While colchicine remains the standard therapy to prevent inflammatory attacks and complications such as amyloidosis, about 5–10% of patients show an inadequate response, and in such cases, anti-IL-1 therapies have emerged as an effective and safe treatment option [4–8].

Given the limited data regarding the duration of these therapies and whether dosing intervals can be reduced, in 2020, our group conducted a Delphi study to reach a consensus on the management of anti-IL-1 therapies, which included recommendations on extending dosing intervals [9]. According to this consensus-based protocol, patients who remain attack-free and free of subclinical inflammation for 6 months after initiating biologic therapy may have their dosing intervals doubled, with a potential further extension to triple the interval after 1 year of sustained stability.

Following this, our group published a retrospective study evaluating patients whose canakinumab dosing intervals were extended according to this protocol [10]. However, follow-up period of these patients was relatively low. This study aimed to present the long-term follow-up data of these patients, providing insights into the effectiveness of the extended dosing regimen.

## Methods

### Patients and study design

The study included 45 pediatric FMF patients who were regularly followed up, had started canakinumab treatment due to colchicine-resistant FMF, and had their canakinumab dosing intervals extended according to the previously described protocol [10]. All patients fulfilled at least one of the diagnostic criteria for FMF, either Eurofever/Paediatric Rheumatology International Trials Organization (PRINTO) 2019 or Tel-Hashomer criteria [11, 12]. The study excluded patients who initiated canakinumab treatment but did not undergo dose interval extension as outlined in the protocol. All included patients were also receiving colchicine treatment concomitantly with canakinumab. In line with the recommendations of the aforementioned Delphi study, all patients were followed at 3-month intervals. At each visit, in addition to clinical evaluation and assessment of disease flares, autoinflammatory disease activity index (AIDAI) scores were calculated, and laboratory parameters in attack-free period were recorded, including complete blood count, liver and kidney function tests, acute phase reactants (CRP, ESR and SAA) and urinalysis. Patient files were reviewed to collect demographic and clinical data, including the attack features, and AIDAI scores at baseline (before anti-IL-1 therapy initiation) and at 6, 12, 18, 24 and 36 months following the start of canakinumab [13]. Additionally, whether the patient had used another biologic agent (anakinra) before canakinumab, and if so, the duration of its use, as well as the duration of colchicine use before starting canakinumab, were recorded. Evidence of subclinical inflammation was also evaluated in the patients, which was defined as elevation above cut-off values in at least one acute phase reactant—CRP (>5 mg/l), SAA (>10 mg/l) or ESR (>20 mm/h)—during attack-free periods, not attributable to other causes such as viral infections. During attacks, patients were clinically and laboratory evaluated, and potential triggers such as viral infections were ruled out. Definitions regarding FMF attacks and colchicine resistance can be found in the previous report [10].

To evaluate the factors influencing the extension of canakinumab dosing intervals, the baseline clinical characteristics, attack features and AIDAI scores of patients who experienced attacks during the protocol or were unable to continue the extended dosing interval due to subclinical inflammation (excluding patients who experienced attacks after canakinumab discontinuation) were compared with those of patients who had no attacks or subclinical inflammation during the follow-up period.

### Statistical analysis

Analysis was performed using Microsoft Excel (Microsoft Corporation, Redmond, WA) for data collection and SPSS 17.0 (IBM, Armonk, NY) for processing. For each variable, descriptive statistics—such as mean, median, interquartile range (IQR) and minimum and maximum—were calculated based on distribution characteristics. Comparisons of categorical variables were performed using the Pearson *χ*^2^ test or Fisher’s exact test. Continuous variables were compared with either the Student’s *t* test or the Mann–Whitney U test, depending on their distribution. Statistical significance was defined as a *p*-value <0.05.

### Ethics

The Ethics Committee of Istanbul University, Istanbul Faculty of Medicine approved this study (approval date and number: 21/05/2020/19).

## Results

A total of 45 patients who began canakinumab treatment on a monthly schedule were enrolled in the study, with dosing intervals extended to every 2 months after the initial 6 months. The median follow-up time from the start of canakinumab therapy across the cohort was 47 months (ranging from 35 to 78 months). The *MEFV* sequence variants were as follows: 40 patients were homozygous for p.(Met694Val), 3 patients were compound heterozygous for p.(Met694Val)/p.(Met680Ile), 1 patient had a compound heterozygous variant of p.(Met680Ile)/p.(Val726Ala) and 1 patient was heterozygous for p.(Met694Val). Baseline demographic and clinical features of the cohort can be found in our previous report.

**Figure 1.**
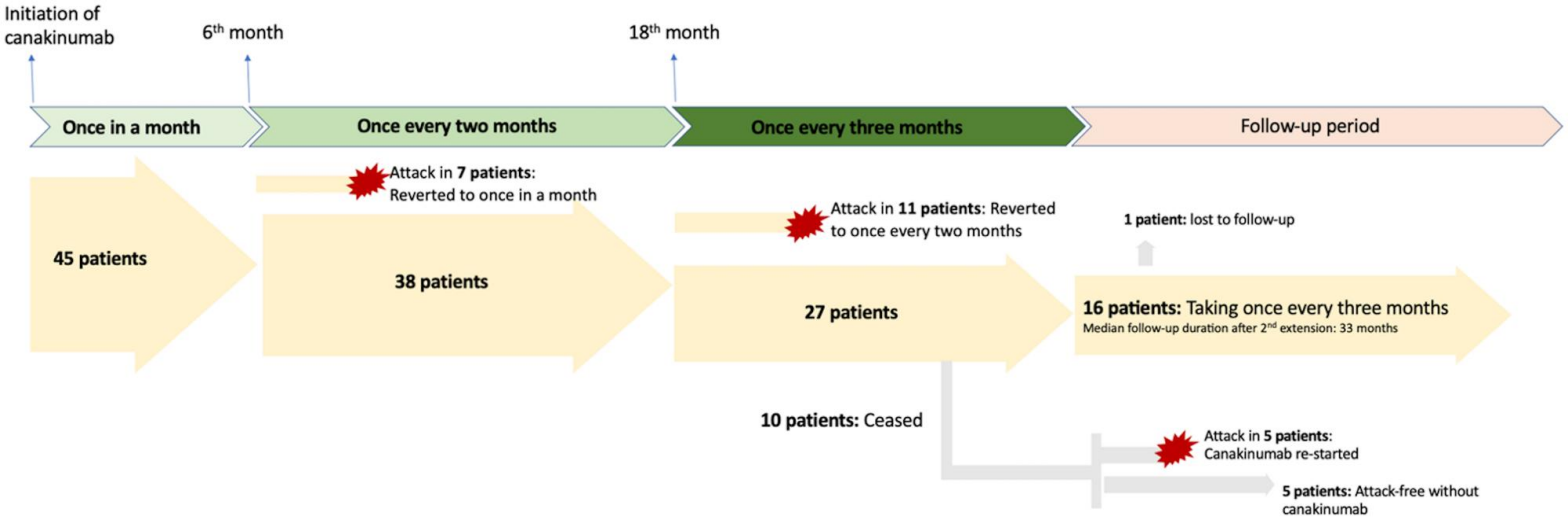
Current dosing schedule summary according to the extension protocol

Following the initial dose interval extension, 7 of these patients (15.56%) had experienced FMF attacks, and their canakinumab dosing was reverted back to monthly. Among the remaining 38 patients (84.44%), the canakinumab dosing interval was further extended to every three months following one year of the first extension. However, in 11 of these patients, dosing had to be reverted to every 2 months because of either clinical attacks or the detection of subclinical inflammation. Among the 27 patients who continued the protocol with a 3-month dosing interval, it was observed that canakinumab was discontinued in 10 patients due to the achievement of long-term remission. Following discontinuation, 5 patients remained attack-free, while the other 5 experienced attacks, necessitating the resumption of therapy. Seventeen patients are still receiving canakinumab every three months and are being followed without attacks or subclinical inflammation, but 1 patient was lost to follow-up, leaving 16 patients (35.56%) who currently continue this regimen. The median follow-up duration after the second dose extension for this group is 33 months (IQR 6.5 months). The dosing schedule and patient progression of the study cohort are presented in Fig. 1.

When comparing the characteristics of patients who were unable to continue the protocol due to experiencing attacks and those who did not experience any attacks during the protocol, no significant differences were found in terms of clinical findings during attacks, baseline monthly attack frequency, attack duration, baseline AIDAI scores, prior use of other anti-IL-1 treatments (if any, and the duration of use), the duration of colchicine use before canakinumab, and baseline laboratory values. However, it was observed that none of the patients with splenomegaly were able to continue with the protocol (*p* = 0.006). Of note, splenomegaly detected on physical examination was further evaluated by ultrasound to rule out other causes such as mass lesions. In all patients, it regressed following anti-IL-1 therapy, as confirmed by follow-up imaging. No accompanying proteinuria was observed. It was not considered a sign of amyloidosis but rather attributed to persistent inflammation, noting that patients with amyloidosis had already been excluded from the study. A family history of amyloidosis was present in four patients (none involved first-degree relatives). Among them, one remained relapse-free on a 3-month regimen, two experienced relapses during extended dosing intervals and returned to monthly treatment, and one relapsed after treatment discontinuation. Due to the small number of cases, no statistical analysis was performed. Additionally, although not statistically significant, the median baseline CRP level was higher in patients who experienced flares during the protocol period compared with those who did not (47.5 [IQR: 59] vs 18.5 [IQR: 52]; *p* = 0.057) (Table 1).

**Table 1.** Comparison of demographic, clinical and laboratory features between patients with and without attacks or subclinical inflammation during the protocol period

		No attacks or subclinical inflammation during the protocol period	Presence of attacks or subclinical inflammation during the protocol period	*p* value
Demographic features				
Age at diagnosis (years)	median (IQR)	3.66 (5.29)	3.29 (5.43)	0.662
Female	*n* (%)	7 (33.3)	10 (55.6)	0.163
Family history of FMF	*n* (%)	15 (71.4)	11 (61.1)	0.496
Family history of amyloidosis	*n* (%)	1 (4.8)	2 (11.1)	0.586^a^
Clinical findings during attacks				
Fever	*n* (%)	21 (100)	18 (100)	
Abdominal pain	*n* (%)	21 (100)	18 (100)	
Pleuritis	*n* (%)	7 (33.3)	5 (27.8)	0.709
Pericarditis	*n* (%)	1 (4.8)	2 (11.1)	0.586^a^
Arthritis	*n* (%)	11 (52.4)	9 (50.0)	0.882
Erysipelas-like erythema	*n* (%)	7 (33.3)	4 (22.2)	0.442
Clinical characteristics				
Protracted febrile myalgia	*n* (%)	1 (4.8)	2 (11.1)	0.586^a^
Anaemia	*n* (%)	6 (28.6)	8 (44.4)	0.303
Splenomegaly	*n* (%)	0 (0)	6 (33.3)	0.006^a^
Growth delay	*n* (%)	1 (4.8)	3 (16.7)	0.318^a^
Anakinra use before canakinumab	*n* (%)	11 (52.4)	10 (55.6)	0.843
Duration of colchicine treatment before canakinumab (months)	Mean (STD)	63.48 (47.88)	81.6 (52.1)	0.264
Duration of anakinra use before canakinumab (months)	Median (IQR)	3 (6)	3.5 (5)	0.972
Baseline number of attacks in the last 6 months before anti-IL-1 treatment^b^	Median (IQR)	12 (10)	6 (6)	0.288
Duration of attacks before anti-IL-1 treatment (days)^b^	Median (IQR)	3.00 (1)	3.00 (1)	0.754
Baseline AIDAI score before anti-IL-1 treatment^b^	Median (IQR)	16.5 (25)	15.5 (12)	0.725
Laboratory values before anti-IL-1 treatment^b^				
Wbc (/µl)	Mean (STD)	10605.0 (3321.6)	8909 (3350.09)	0.108
Hb (g/dl)	Mean (STD)	11.7 (1.50)	11.8 (1.34)	0.846
ESR (mm/h)	Median (IQR)	32 (51)	28.5 (32)	0.918
SAA (mg/dl)	Median (IQR)	14.0 (18)	15.2 (367)	0.577
CRP (mg/l)	Median (IQR)	18.5 (52)	47.5 (59)	0.057

AIDAI, autoinflammatory disease activity index; CRP, C-reactive protein; ESR, erythrocyte sedimentation rate; FMF, familial Mediterranean fever; Hb, haemoglobin; IL-1, interleukin-1; IQR, interquartile range; SAA, serum amyloid A; STD, standard deviation; WBC, white blood cell count.

a Fisher’s exact test.

b Before the initiation of the first biologic agent (anakinra or canakinumab).

Due to the low number of patients with genotypes other than p.(Met694Val) homozygosity, genotype-specific statistical analysis could not be performed. Treatment response among these patients appeared variable. Among those with the p.(Met694Val)/p.(Met680Ile) genotype (*n* = 3), one relapsed after treatment discontinuation, one remained attack-free during follow-up, and one was lost to follow-up. The patient with the p.(Met680Ile)/p.(Val726Ala) genotype had no relapses, while the heterozygous p.(Met694Val) patient experienced a relapse while on a 2-month dosing interval.

## Discussion

This study evaluated the long-term outcomes of a large pediatric FMF cohort in which canakinumab dosing intervals were extended according to a standardized protocol. Only patients managed according to the standardized follow-up protocol (monthly for 6 months, every 2 months for 1 year, then every 3 months) were included, as per Delphi-based consensus. Given the retrospective design and the heterogeneity of previous evaluations, focusing on a uniform protocol ensured more reliable outcome assessment. Our findings demonstrate that this protocol is promising and may serve as a practical approach to optimize treatment in selected patients. Given the limited data in the current literature on long-term dose extension of canakinumab, we believe our results contribute valuable insights.

In our previous study focusing on short-term outcomes, we observed that patients who remained attack-free after transitioning to dosing every 2 months were generally able to tolerate a 3-month dosing interval. Only one patient had an attack at this phase [10]. However, in the present study, which includes long-term follow-up, 11 out of 38 patients experienced attacks after the dosing interval was increased to 3 months, during a median follow-up period of 33 months. These findings indicate that although most patients can tolerate quarterly dosing over the long term, a subset may be at risk of disease flares and may require closer monitoring.

The protocol did not include complete discontinuation of canakinumab. Nevertheless, due to the retrospective nature of the study, treatment was discontinued in 10 patients during follow-up. As these cases deviated from the defined protocol, they were evaluated separately.

Pharmacokinetic studies have shown that canakinumab has a half-life of ∼22 to 26 days [14]. Therefore, a 3-month dosing interval can be considered relatively low-intensity treatment. Importantly, some patients who remained stable on the 3-month dosing regimen experienced flares after complete discontinuation, underscoring the continued therapeutic effect of canakinumab even at extended intervals in some individuals. Conversely, a few patients maintained remission after complete discontinuation, suggesting that long-term anti-IL-1 therapy may not be necessary for all patients. However, our study was not designed to evaluate treatment discontinuation, and although the generalizability of these observations is limited, the data support that a 3-month dosing interval may be sufficient to maintain disease control in some cases.

Data on dose tapering and discontinuation of anti-IL-1 therapies are primarily derived from pediatric studies, and encouraging results have been reported with such approaches [15, 16]. The underlying mechanisms of colchicine resistance and persistent hyperinflammation in FMF patients are not yet fully understood. However, in clinical practice, it is often observed that some children considered colchicine-resistant regain responsiveness following anti–IL-1 therapy. We hypothesize that this may be attributed to potentially transient pro-inflammatory stimuli—such as infections—that are more frequently encountered during childhood and may induce a reversible state of colchicine unresponsiveness. Nonetheless, since all patients met the established criteria for colchicine resistance, distinguishing those influenced by such transient factors remains challenging. Consistent with this, we were unable to identify any clear distinguishing features—aside from splenomegaly—between patients who experienced disease flares during canakinumab tapering and those who remained attack-free. Additionally, although not statistically significant, baseline (before anti-IL-1 therapy) CRP levels tended to be higher in patients who experienced flares during the protocol. This may reflect a link between CRP and long-standing persistent inflammation, and suggests that baseline CRP could potentially serve as an additional parameter to consider when deciding on dose interval extension.

Although this protocol appears promising, it is essential to emphasize that decisions regarding dose interval extension or treatment discontinuation should be guided by the treating physician’s expertise, based on comprehensive patient evaluation and continuous monitoring. In this context, close physician–patient communication remains critical to minimizing the risk of adverse outcomes, such as amyloidosis, that may result from inadequate disease control. Although a recent large cohort study from Turkey reported a low amyloidosis rate, this issue should be especially emphasized in patients with a family history of amyloidosis, who require closer monitoring [17]. Furthermore, in clinical situations that require individualized decision-making, ensuring that healthcare policies—such as treatment reimbursement or prescribing regulations—allow sufficient flexibility may be important to support physician autonomy and maintain optimal treatment adherence.

The limitation of our study lies in its retrospective design, which naturally restricts our ability to determine cause-and-effect relationships. However, we believe that the large sample size, use of a standardized protocol, and the long follow-up duration provide significant value to the findings. Despite these strengths, it is clear that prospective and interventional studies are necessary to better confirm the applicability of standardized protocols.

In conclusion, this study demonstrates that extending canakinumab dosing intervals in a pediatric FMF cohort according to a standardized protocol is a promising approach to optimize treatment. While most patients tolerated the extended dosing interval without flare-ups, a subset experienced attacks, highlighting the need for closer monitoring. Although the study’s retrospective nature limits definitive conclusions, our findings suggest that canakinumab remains effective even with less frequent dosing. Prospective and interventional studies are needed to further validate the long-term applicability for this dosing strategy.

## Data Availability

The datasets of the current study are available from the corresponding author on reasonable request.
